# Universal and specific quantitative detection of botulinum neurotoxin genes

**DOI:** 10.1186/1471-2180-10-267

**Published:** 2010-10-20

**Authors:** Brenna J Hill, Janet C Skerry, Theresa J Smith, Stephen S Arnon, Daniel C Douek

**Affiliations:** 1 Human Immunology Section, Vaccine Research Center, National Institutes of Allergy and Infectious Diseases, National Institutes of Health, Bethesda, MD 20892 USA; 2 Integrated Toxicology Division, USAMRIID, Fort Detrick, MD 21702 USA; 3 Infant Botulism Treatment and Prevention Program, California Department of Public Health, Richmond, CA 94804 USA

## Abstract

**Background:**

*Clostridium botulinum*, an obligate anaerobic spore-forming bacterium, produces seven antigenic variants of botulinum toxin that are distinguished serologically and termed "serotypes". Botulinum toxin blocks the release of acetylcholine at neuromuscular junctions resulting in flaccid paralysis. The potential lethality of the disease warrants a fast and accurate means of diagnosing suspected instances of food contamination or human intoxication. Currently, the Food and Drug Administration (FDA)-accepted assay to detect and type botulinum neurotoxins (BoNTs) is the mouse protection bioassay. While specific and sensitive, this assay requires the use of laboratory animals, may take up to four days to achieve a diagnosis, and is unsuitable for high-throughput analysis. We report here a two-step PCR assay that identifies all toxin types, that achieves the specificity of the mouse bioassay while surpassing it in equivalent sensitivity, that has capability for high-throughput analysis, and that provides quantitative results within hours. The first step of our assay consists of a conventional PCR that detects the presence of *C. botulinum *regardless of the neurotoxin type. The second step uses quantitative PCR (qPCR) technology to determine the specific serotype of the neurotoxin.

**Results:**

We assayed purified *C. botulinum *DNA and crude toxin preparations, as well as food and stool from healthy individuals spiked with purified BoNT DNA, and one stool sample from a case of infant botulism for the presence of the NTNH gene, which is part of the BoNT gene cluster, and for the presence of serotype-specific BoNT genes. The PCR surpassed the mouse bioassay both in specificity and sensitivity, detecting positive signals in BoNT preparations containing well below the 1 LD_50 _required for detection via the mouse bioassay. These results were type-specific and we were reliably able to quantify as few as 10 genomic copies.

**Conclusions:**

While other studies have reported conventional or quantitative PCR-based assays for the detection of *C. botulinum *genes, our procedure's high-throughput capability and its portability allows most laboratories to quickly assess the possible presence of BoNTs either in food processing samples or in suspected cases of botulism. Thus, this assay provides rapid and specific detection of BoNT and toxin complex genes and would enable the targeting of appropriate therapeutics to infected individuals in a timely manner.

## Background

*Clostridium botulinum*, an obligate anaerobic spore-forming bacterium, produces botulinum neurotoxin (BoNT), the most potent toxin known [1-3]. BoNT is classified as a Category A biothreat agent by the Centers for Disease Control and Prevention (CDC) because of its lethality and ease of production, transport and dissemination [4,5]. In addition, BoNT poses several threats to the public health: first, the possibility of foodborne botulism represents a major potential health hazard that requires continual monitoring by the food industry. Second, infant botulism has been the most common form of human botulism in the United States for more than 20 years and hospitalizes approximately 80-100 U.S. infants annually [6]. Third, cases of wound botulism due to intravenous drug use continue to increase [7,8].

Botulism toxicity results from one of seven serologically distinct neurotoxins (types A-G) that cause a severe neuroparalytic disease characterized by descending flaccid paralysis [9]. Rarely, unique strains of *C. butyricum and C. baratii *may also cause human botulism through production and release of BoNT/E and F, respectively [10,11]. The toxin acts by binding to peripheral cholinergic nerve endings and inhibiting release of acetylcholine at the neuromuscular junction. A part of the toxin is a zinc-dependent protease that cleaves target substrate proteins (SNAREs), located either on the plasma membrane or the synaptic vesicle, thereby preventing their binding, fusion and release of neurotransmitter. BoNTs cleave specific amino acids on the target proteins of the SNARE complex. BoNT/A and BoNT/E act on SNAP-25, while BoNT/C targets syntaxin as well as SNAP-25. The remaining toxin types (BoNT/B, BoNT/D, BoNT/E and BoNT/F) all act on synaptobrevin, but at different cleavage sites [12-15].

The potential severity and lethality of the disease warrants sensitive and specific detection and serotyping of toxin and its typing to enable correct administration of serotype-specific antitoxin in a timely manner. Although treatment with Human Botulism Immune Globulin (BabyBIG^®^) or equine antitoxin is based on clinical findings and should be instituted as rapidly as possible [5,16,17], definitive microbiological diagnosis may take several days or even longer. This extended time to diagnosis occurs because detection of the bacterium and its toxin relies on toxicity assessment in mice (the mouse protection bioassay) and lengthy culture assays, which, while sensitive and specific, may be time-consuming and difficult [18,19]. Moreover, the availability of the mouse protection bioassay is limited due to lack of animal facilities and reagent constraints.

A readily available rapid diagnostic test would be valuable for public health and medical management of foodborne, infant, wound, or bioterrorist botulism outbreaks. Quick, accurate diagnosis would enable the limited supply of equine or human antitoxin to be directed to affected patients, thereby allowing exposed but unaffected individuals to be reassured and spared unnecessary treatment with an equine serum product. A high-throughput assay would also be beneficial to the food industry, where the use of large quantities of mice is impractical.

Several studies have described PCR-based assays that detect the various serotypes of BoNT genes [20-26]. With the advent of quantitative PCR (qPCR), further studies have reported assays that detect the toxin types (A, B, E and F) generally implicated in human illness and food contamination [27-31]. However, comprehensive sequence analysis shows a high level of genetic variability within the toxin types that enables differentiation of toxin types into subtypes [32,33]. Thus, existing assays may not reliably detect all known subtype variants within each botulinum toxin type.

For these reasons we have developed a novel two-step PCR-based assay that can detect both BoNT and other gene sequences located within the toxin gene complex. It is known that *C. botulinum *DNA is readily attracted to botulinum neurotoxins, necessitating the use of various treatments for the removal of nucleic acids during toxin purification [34-37]. These DNA sequences may be found even in highly purified protein preparations of the toxin and are therefore a reliable surrogate for the presence of BoNT, enabling rapid detection without using mice. As antitoxin doses are administered based on the serotype of toxin and clinical symptoms and not on the amount of active toxin present in the sample, the assay described here will provide the critical information needed for clinicians to treat affected patients. The first step in this procedure is a universal electrophoresis-based PCR that detects the presence of the *C. botulinum *nontoxin-nonhemagglutinin (NTNH) gene, a highly conserved toxin complex gene that is found in all *C. botulinum *toxin types and subtypes that has been found in all BoNT-producing *C. botulinum *gene sequences examined to date [32,38]. Thus, samples that contain BoNT can be identified irrespective of serotype, thereby providing comprehensive but not type-specific detection. A similar independent assay to detect NTNH has recently been reported by Rafael and Andreadis [38]. The second step of the assay uses qPCR to determine quantitatively the specific BoNT toxin type by using seven different degenerate primer/probe pairs, one for each of the seven A-G toxin serotypes. These assays successfully detected toxin genes from 22 of the 26 known toxin subtypes.

## Results

### Universal detection of the *C. botulinum *toxin complex gene NTNH

**Figure 1:**
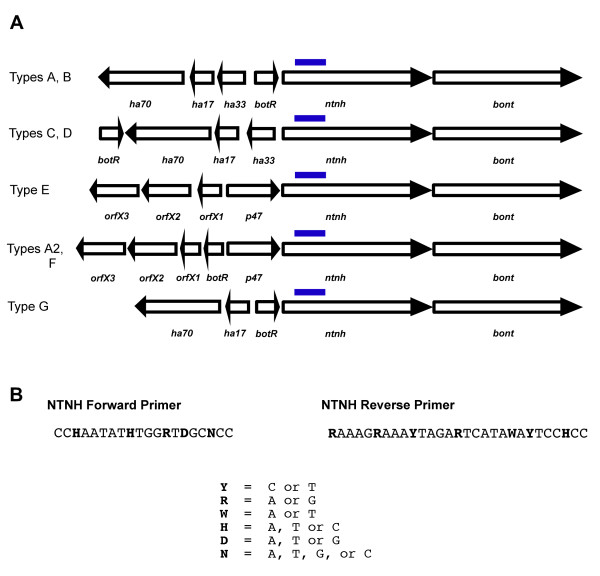
**Selection and design of universal PCR primers**. (A) Diagram of *C. botulinum *neurotoxin gene (BoNT) organization (adapted from Chen et al. 2007) [39]. (B) Non-toxin non-hemagglutinin gene (NTNH) primers targeting a highly conserved area directly upstream from BoNT. Primer sequences contain degenerate bases to accommodate all strain variation.

Figure 1A shows the *C. botulinum *neurotoxin complex gene organization for each type [39,40] and the primers designed for the nontoxin-nonhemagglutinin gene (NTNH) (Figure 1B), which is present in all *C. botulinum *types directly upstream from the neurotoxin gene in BoNT toxin gene clusters. The primers target an area that is highly conserved between *C. botulinum *types A-G. Degenerate primers were designed to accommodate any base discrepancy in the target area.

We tested these primers with DNA purified from *C. botulinum *cultures of each toxin type and also included control genomic and plasmid DNA from samples of *E. coli *bacterial colonies (DH5α) as well as crude lysate from human peripheral blood mononuclear cells. A specific NTNH product of 101 base pairs was detected in each lane containing clostridial DNA representing all toxin serotypes as well as BoNT-producing *C. butyricum *and *C. baratii *isolates, but no band was detected in any of the controls.

We also confirmed that detection of the NTNH gene was specific to BoNT-producing clostridial species. Table 1 shows the results of the universal PCR performed with DNA purified from clostridial species harbouring the BoNT gene and those lacking these genes. A strong PCR product was detected from all samples that expressed detectable levels of BoNTs, but not from any clostridial strain that did not produce BoNTs.

**Table 1:** NTNH gene detection on *C. botulinum *and other clostridial strains

BoNT subtype	strain	PCR(DNA)^a^	(culture supernatant)^b^	other clostridia	strain	PCR(DNA)^a^
A1	Hall	+	+	C. absonum	ATCC 27555	-
A1	CDC 1757	+	+	C. baratii ^e^	ATCC 27638	-
A1	CDC 1744	+	+	C. bifermentans	ATCC 638	-
A2	Kyoto-F	+	+	C. haemolyticum	ATCC 9650	-
A2b	CDC 1436	+	+	C. hastiforme	ATCC 25772	-
A3	Loch Maree	+	+	C. histolyticum	ATCC 19401	-
B1	Okra	+	+	C. novyi	ATCC 17861	-
B1	CDC 1656	+	+	C. novyi	ATCC 19402	-
B1	CDC 1758	+	+	C. novyi A	ATCC 19402	-
B2	213B	+	+	C. novyi B	ATCC 2706	-
B2	CDC 1828	+	+	C. perfringens A	ATCC 3624	-
B4 (npB)	Eklund 17B	+	+	C. perfringens A	ATCC 12915	-
Ba4	CDC 657	+	+	C. perfringens A	ATCC 12917	-
Bf	An436	+	+	C. perfringens A	ATCC 12918	-
C	Stockholm	+	-	C. perfringens A	ATCC 12919	-
C/D	6813	+	-	C. perfringens A	ATCC 13124	-
D	ATCC 11873	+	+	C. perfringens B	ATCC 3626	-
D	1873	+	nd	C. perfringens D	ATCC 3629	-
D/C	VPI 5995	+	+	C. perfringens D	ATCC 3630	-
E1	Beluga	+	-	C. perfringens D	ATCC 3631	-
E2	CDC 5247	+	nt	C. perfringens D	ATCC 12920	-
E2	CDC 5906	+	nt	C. perfringens E	ATCC 27324	-
E3	Alaska E43	+	+	C. ramosum	ATCC 25582	-
E4 (It butyr)^c^	BL5262	+	-	C. septicum	ATCC 12464	-
F1 (prot)	Langeland	+	+	C. sordelli	ATCC 9714	-
F2 (np)	Eklund 202F	+	-	C. sporogenes	ATCC 19404	-
F3 (baratii)^d^	Orange	+	nt	C. sporogenes	ATCC3854	-
G	1354	+	nd	C. subterminale	ATCC 25774	-
				C. tertium	ATCC 14573	-
				C. tetani	ATCC 10799	-
				C. tetani	ATCC19406	-

^a ^+/- indicates presence/absence of 101 bp band on agarose gel. Samples are purified DNA from bacterial cultures as described in the Methods section.

^b ^Samples originate from filtered culture supernatants containing crude toxin. +/- indicates presence/absence of 101 bp band on agarose gel. nd = not detected, nt = not tested.

^c ^BoNT E-producing strain of *C. butyricum *isolated from an infant case in Italy.

^d ^BoNT F-producing strain of *C. baratii*.

^e^Non-toxin producing strain of *C, baratii*.

Results from conventional PCR detection of NTNH. A (+/-) indicates presence/absence of 101 bp band by agarose gel, respectively. DNA results indicate PCR detection of NTNH in purified DNA from both *C botulinum *and other *Clostridial *strains. Culture supernatant results indicate amplification of DNA within crude culture supernatants. NT indicates samples that were not tested.

We next confirmed the robustness of NTNH detection both on food samples that were spiked with purified serotype-specific *C. botulinum *DNA and on crude toxin preparations. Canned vegetables and canned meat were spiked with 100 μL of purified DNA at dilutions down to 1 genomic copy of type-specific BoNT DNA in 100 μL. DNA was extracted from spiked samples as described in the methods section. Only samples that had been spiked with clostridial DNA from neurotoxin-containing strains tested positive for NTNH (data not shown). As with the food samples, DNA was extracted from crude toxin-containing cultures and tested for the presence of NTNH. All of the purified DNA samples and most of the crude culture supernatant samples examined were positive for NTNH (Table 1). The lack of amplification from some of the crude culture supernatants may be due to lack of DNA extraction resulting in the presence of proteinaceous PCR inhibitors.

In addition to spiking food, we also spiked healthy infant stool with varying concentrations of BoNT serotype-specific *C. botulinum *DNA as described in the materials and methods. We detected a positive PCR result in all samples of stool spiked with BoNT DNA to an amount as low as an equivalent of 10 genomic copies. In the sample spiked with BoNT A at an equivalent of 1 genomic copy, we obtained a weak positive PCR result. Additionally, we tested DNA extracted from a clinical sample from a recent case of infant botulism, diagnosed by the mouse protection bioassay, and clearly detected presence of the NTNH gene (Table 2).

**Table 2:** NTNH PCR detection in spiked healthy infant stool and a clinical infant botulism sample

Strain (genomic copies)	NTNH Detection
BoNT A (10^4^)	++
BoNT A (10^2^)	++
BoNT A (10)	+
BoNT A (1)	+
	
BoNT B (10^4^)	++
BoNT B (10^2^)	+
BoNT B (10)	+
BoNT B (1)	-
	
BoNT C (10^4^)	++
BoNT C (10^2^)	+
BoNT C (10)	+
BoNT C (1)	-
	
BoNT D (10^4^)	++
BoNT D (10^2^)	+
BoNT D (10)	-
BoNT D (1)	-
	
BoNT E (10^4^)	++
BoNT E (10^2^)	+
BoNT E (10)	+
BoNT E (1)	-
	
BoNT F (10^4^)	++
BoNT F (10^2^)	++
BoNT F (10)	+
BoNT F (1)	-
	
BoNT G (10^4^)	++
BoNT G (10^2^)	++
BoNT G (10)	+
BoNT G (1)	-
	
Clinical stool sample	++

++ Denotes strong PCR band

+ Denotes weak PCR band

- Denotes no PCR Band

Listed in this table are results from NTNH gene detection within spiked samples of a healthy infant stool sample as described in the materials and methods. Also included is the result from a confirmed case of infant botulism in California. (++) indicates a strong positive PCR product at the dilution tested, (+) is a weak positive PCR product, and (-) indicates no amplification detected.

### Quantitative type-specific detection of *C. botulinum*

**Figure 2:**
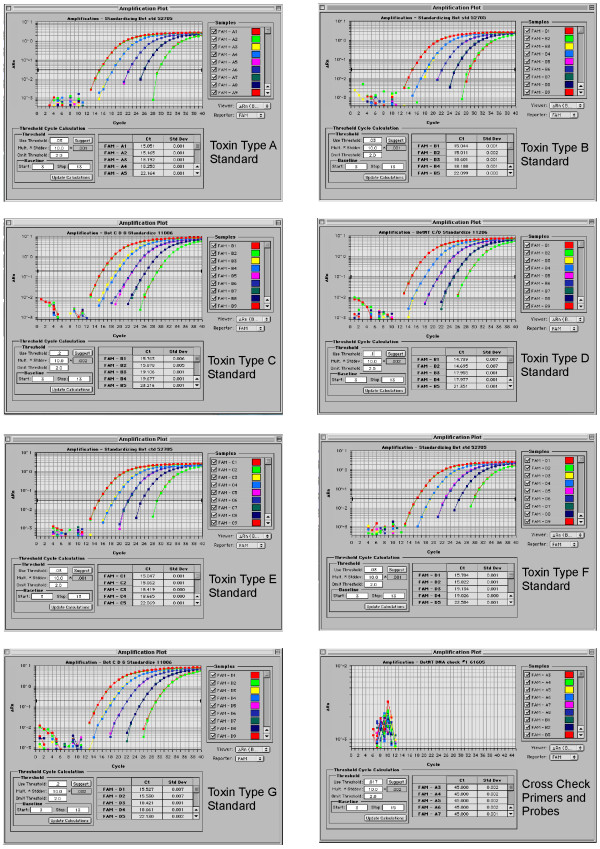
**qPCR validation of plasmid standards**. Each standard dilution tested against type-specific primers and probes and cross-checked with primers and probes specific to all remaining types. Five ten-fold serial dilutions tested with each appropriate primer and probe set are depicted in each amplification plot. All reactions amplified with non-type-specific primer and probe sets show no amplification and are represented in bottom right amplification plot.

We designed primers and probes specific to each toxin type (A-G). Each set targets portions of the light chain of the neurotoxin gene in areas conserved within each subtype yet unique to each toxin type such that no cross-reactivity should occur. Any base differences between strains were accounted for by incorporation of degenerate bases (Table 3). As validation, Figure 2 shows results of the type-specific qPCR performed on the plasmid standards corresponding to each *C. botulinum*. Not only was each primer/probe set able to detect its *C. botulinum *type toxin gene sequence sensitively and specifically, there was also no cross-reactivity of any primer/probe set with a toxin gene sequence from a different *C. botulinum *type.

**Table 3:** Primer and probe sets for each serotype used in quantitative PCR

**Toxin Class**	**Sequence**	**Location on Toxin Gene(bp)**
BoNT A Forward	TGGTTTTGAGGAGTCACTTGAA	582
BoNT A Reverse	TCATGTCCCCCAAATGTTCT	809
BoNT A Probe	TGCAGGCAAATTTGCTACAGATCCA	627
BoNT B Forward	CAAGAAAACAAAGGCGCAAG	619
BoNT B Reverse	CTGGGATCTTGYCCTCCAAA	833
BoNT B Probe	CGTGGATATTTTTCAGATCCAGCCTTG	652
BoNT C Forward	CAACTTTAATTATTCAGATCCTGTTGA	18
BoNT C Reverse	GGCTTGTAACTCGAGGAGGTT	199
BoNT C Probe	TGAGCCTGAAAAAGCCTTTCGCA	93
BoNT D Forward	CCATCATTTGAAGGGTTTGG	541
BoNT D Reverse	TGGGTCCATCTTGAGARAAA	791
BoNT D Probe	GATTCGTCCACAAGTTAGCGAGGGA	744
BoNT E Forward	ATAATGGGAGCAGAGCCTGA	448
BoNT E Reverse	CCCTTTAGCCCCATATAGTCC	678
BoNT E Probe	TGCCAAGCAATCACGGTTTTGG	515
BoNT F Forward	GTSAGACAATACCTCAAATATCAAATCG	1488
BoNT F Reverse	CTGGYACTTTTTGTGCATGT	1646
BoNT F Probe	TGCCAAGATATGATTCTAATGGAA	1551
BoNT G Forward	ATCCAACCTGGAGCTGAAGA	427
BoNT G Reverse	GCTGGATCTGCAAAATACGC	674
BoNT G Probe	TGGCCATTCCCCAATATCAGAAGG	534

**Y **= C or T

**R **= A or G

**S **= G or C

Indicated in this table are the type specific primers and probes for each BoNT tested in this manuscript. Included are forward, reverse and probe sequences and their locations within the toxin gene. Bases indicated in bold represent degenerate bases: **Y **represents C or T; **S **represents C or G, and **R **represents A or G.

**Figure 3:**
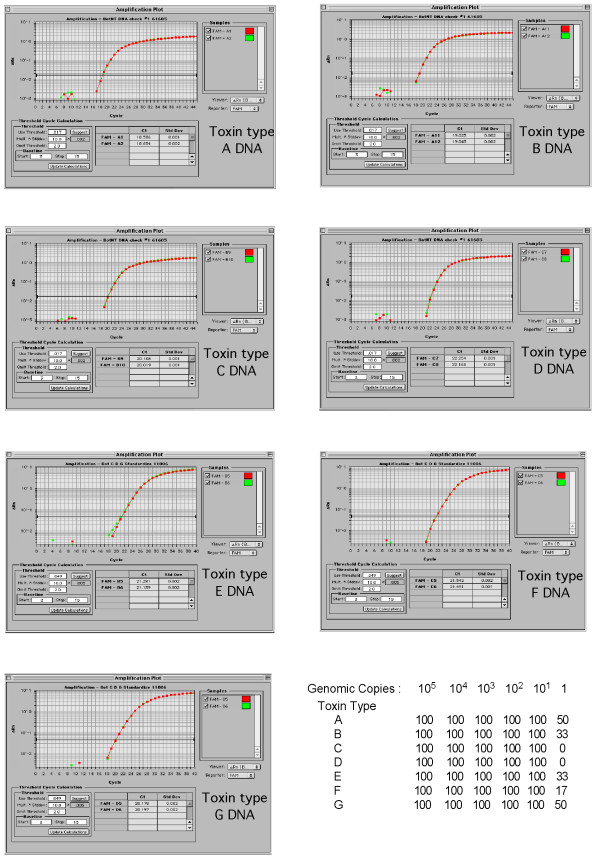
**qPCR detection of type-specific neurotoxin DNA**. Each toxin type DNA amplified with type-specific primers and probes. Assay sensitivity is shown in the table. Each toxin type DNA was amplified with its cognate primer and probe set. The DNA was diluted based on its concentration and genomic size such that each reaction contained a known number of DNA target gene copies. Dilutions ran from 10^5 ^genomic copies to 1 genomic copy. Each dilution series was run with six replicates to determine reproducibility. Plasmid standards were amplified along with each dilution series to determine exact copy number in each reaction. Results represent the percentage of the six replicates that contained accurate copy numbers in each reaction.

Figure 3 shows quantitative type-specific amplification of DNA purified from laboratory-cultured samples of *C. botulinum *representing all toxin types A-G. Each primer/probe set amplified only that DNA of the specific toxin gene type with no amplification of toxin gene sequences of a differing type. As confirmation of our assay, we diluted purified DNA from *C. botulinum *cultures taking into account genomic size and concentration of the DNA preparation. We made 5 ten-fold dilutions representing 10^5 ^to one genomic copies of BoNT and tested six replicate reactions per assay. Figure 3 (table) shows that the sensitivity of detection is consistently as low as 10 gene copies per reaction. Using our plasmid standards, actual values consistently showed accurate target gene copy numbers within each dilution and were reproducible in each replicate reaction. We were able to detect 1 copy of the BoNT gene in several toxin samples, but the overall detection level of our assay was reliably as few as 10 copies of neurotoxin gene.

**Figure 4:**
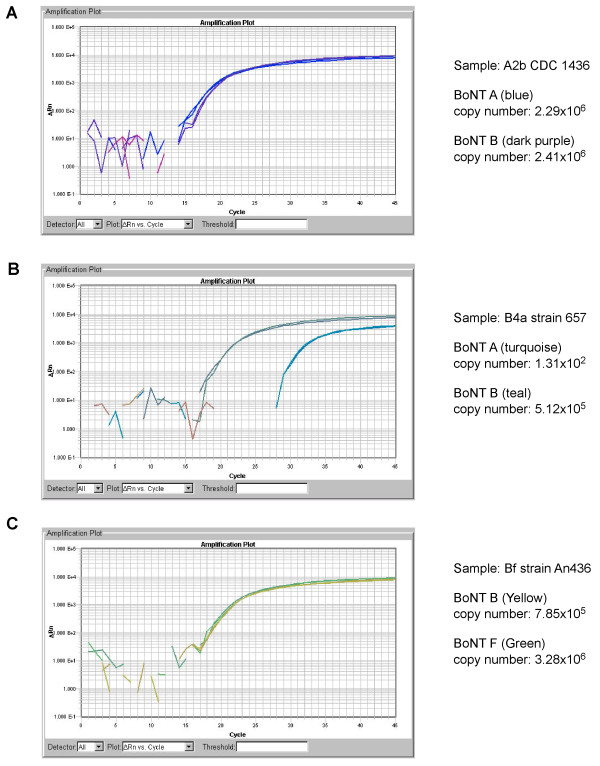
**Detection of silent genes in dual BoNT containing strains of *C. botulinum***. Shown are amplification plots of three strains of *C. botulinum *that contain silent genes: CDC1436 A2b (A), strain 657 Ba4 (B), and strain An436 Bf (C). Copy numbers and the indicated gene detected by color are listed for each.

To confirm the specificity of the assay, we further extracted DNA from pure laboratory-cultures from twenty-nine *C. botulinum *strains representing twenty-two different toxin subtypes. Amplification occurred only when DNA from a particular BoNT serotype was paired with its type-specific primer/probe set, and there was no cross-reactivity between primer/probe sets of one serotype and toxin genes of a different serotype (Table 4). Importantly, strains known to produce or contain the genes for two toxin serotypes were successfully confirmed as such by the assay (Figure 4).

**Table 4:** Cross reactivity and specificity of primers and probes with all subtypes of *C. botulinum *Subtype Specific Primer and Probe set Used in Real Time PCR

BoNT subtype	strain	A	B	C	D	E	F	G
A1	Hall	+	-	-	-	-	-	-
A1	CDC 1757	+	-	-	-	-	-	-
A1	CDC 1744	+	-	-	-	-	-	-
A2	Kyoto-F	+	-	-	-	-	-	-
A2b	CDC 1436	+	+	-	-	-	-	-
A3	Loch Maree	+	-	-	-	-	-	-
B1	Okra	-	+	-	-	-	-	-
B1	CDC 1656	-	+	-	-	-	-	-
B1	CDC 1758	-	+	-	-	-	-	-
B2	213B	-	+	-	-	-	-	-
B2	CDC 1828	-	+	-	-	-	-	-
B3	CDC 795	-	+	-	-	-	-	-
B4 (npB)	Eklund 17B	-	+	-	-	-	-	-
Ba4	CDC 657	+	+	-	-	-	-	-
Bf	An436	-	+	-	-	-	-	-
C	Stockholm	-	-	+	-	-	-	-
C/D	6813	-	-	+	-	-	-	-
D	ATCC 11873	-	-	-	+	-	-	-
D	1873	-	-	-	+	-	-	-
D/C	VPI 5995	-	-	-	+	-	-	-
E1	Beluga	-	-	-	-	+	-	-
E2	CDC 5247	-	-	-	-	+	-	-
E2	CDC 5906	-	-	-	-	+	-	-
E3	Alaska E43	-	-	-	-	+	-	-
E4 (It butyr)	BL5262	-	-	-	-	+	-	-
F1 (prot)	Langeland	-	-	-	-	-	+	-
F2 (np)	Eklund 202F	-	-	-	-	-	+	-
F3 (baratii)	Orange	-	-	-	-	-	+	-
G	1354	-	-	-	-	-	-	+

"+/-" indicates amplification/no amplification by real time PCR

Indicated in the table are all strains tested with each type-specific BoNT primer and probe set. A (+/-) indicates amplification/no amplification by real time PCR.

**Figure 5:**
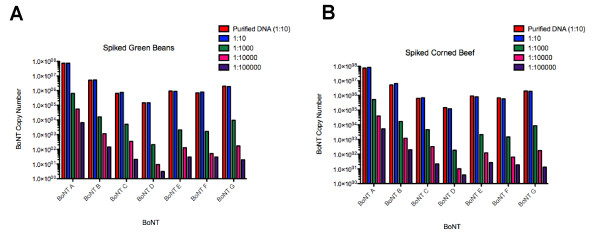
**qPCR detection of type-specific BoNT DNA in food samples spiked with purified *C. botulinum *DNA**. Canned green beans or corned beef was spiked with ten-fold dilutions of purified type-specific BoNT DNA. Samples were processed and DNA extracted from each sample. Results show copy number of each type-specific BoNT dilution in both food types.

We then tested DNA-spiked food samples and crude culture supernatants for the presence of serotype-specific BoNT genes using the above assays. In spiked food samples, we were able to detect type-specific BoNT DNA down to at least three genomic copies of BoNT DNA in each sample (Figure 5A and 5B). To determine relative levels of detections, we tested the four major causes of foodborne botulism, BoNT A, B, E, and F within crude toxin supernatants. Positive PCR signals were seen with sample dilutions containing toxin concentrations of 0.000018 LD_50 _BoNT/A per ml and 0.00385 LD_50 _BoNT/B toxin per ml. The level of detection is greater than 50,000 times more sensitive than the mouse bioassay for BoNT/A and greater than 250 times more sensitive than the mouse bioassay for BoNT/B in equivalent samples. Positive PCR signals were observed with sample dilutions equal to 1LD_50 _in BoNT/E toxin/mL and 0.007 LD_50 _BoNT/F toxin/mL. Thus the level of detection for BoNT/E and BoNT/F matched or was 1000 times more sensitive than the mouse protection bioassay, respectively (Table 5).

**Table 5:** Detection limits of BoNT DNA in crude toxin supernatants

	Bot A	Bot B	Bot E	Bot F
Crude Toxin 2 ng	LOD			
Crude Toxin 200 pg		LOD		LOD
Crude Toxin 20 pg			0.8 (LOD)	
Crude Toxin 2 pg				
Crude Toxin 200 fg		11.7		2.58
Crude Toxin 20 fg	29.2			

LOD indicates the averaged limits of detection for that subtype in our mouse protection bioassay with identical serotypes used in toxin complex preparations.

Indicated in this table is the sensitivity of quantitative PCR for type-specific BoNT detection. Crude toxin supernatants with equivalent toxin protein amounts are listed. Values included in this table are the exact copy number of BoNT DNA detected in crude toxin preparations at the indicated amounts of protein. LOD indicates the averaged limits of detection for that subtype in our mouse protection bioassay with identical serotypes used in toxin complex preparations.

Next, we did comparative testing of crude culture supernatants (without DNA extraction) against purified DNA preparations from the same strains. As the crude culture supernatants contained botulinum neurotoxins, they were tested at an independent location that is registered for the use of botulinum neurotoxins using alternative equipment (the Roche Light Cycler versus the ABI 7700 for the purified DNA preparations). All 23 BoNT-containing samples tested positive for the appropriate toxin subtype, including three samples containing multiple toxin serotypes (A2b, Ba4, and Bf). In addition, the mosaic C/D and D/C strains had positive PCR signals for both serotype C and D, confirming the existence of both BoNT/C and/D gene sequences in these strains. The results, shown in Table 6, indicate that this assay is equally effective at detecting and identifying BoNT genes regardless of the sample (crude culture supernatants or purified DNA preparations) or the equipment used.

**Table 6:** Detection of BoNT DNA from purified DNA of bacterial cultures or extracted DNA from crude toxin supernatants

		BoNT A		BoNT B		BoNT C		BoNT D		BoNT E		BoNT F		BoNT G	
BoNT subtype	strain	ABI	LC	ABI	LC	ABI	LC	ABI	LC	ABI	LC	ABI	LC	ABI	LC
A1	Hall	++++	+++												
A2b	CDC 1436	++	++++		+++										
A3	Loch Maree	++	++++												
B1	Okra			++++	+++										
B2	213B			++++	++										
B2	CDC 1828			++++	+++										
B3	CDC 795			+++	+++										
B4 (npB)	Eklund 17B			++	+++										
Ba4	CDC 657	+	+	+++	+++										
Bf	An436			+++	+++							++	+++		
C	Stockholm					++++	+++								
C/D	6813					++	++	++							
D	ATCC 11873							++	+++						
D/C	VPI 5995					++		++++	+++						
E1	Beluga									++++	++				
E2	CDC 5247									++++	++				
E2	CDC 5906									+++	++				
E3	Alaska E43									++++	+++				
E4 (It butyr)	BL5262									+++	++				
F1 (prot)	Langeland											++++	+++		
F2 (np)	Eklund 202F											+++	++		
F3 (baratii)	Orange											++			
G	1354													++++	+++
C.sporogenes		-	-	-	-	-	-	-	-	-	-	-	-	-	-
	10^6 ^standard	++++	++++	++++	++++	++++	++++	++++	++++	++++	++++	++++	++++	++++	++++
	10^5 ^standard	++++	++++	++++	++++	++++	++++	++++	++++	++++	++++	++++	++++	++++	++++
	10^4 ^standard	+++	+++	+++	+++	+++	+++	+++	+++	+++	+++	+++	+++	+++	+++
	10^3 ^standard	+++	+++	+++	+++	+++	+++	+++	+++	+++	+++	+++	+++	+++	+++
	10^2 ^standard	+++	+++	+++	+++	+++	++	+++	+++	+++	+++	+++	+++	+++	++
	negative	-	-	-	-	-	-	-	-	-	-	-	-	-	-

ABI = Extracted DNA run on an ABI 7700 real time PCR sequence detector.

LC- = Crude *C. botulinum *culture supernatants run on the Roche Light Cycler. 0-20 cycles = ++++,

21-30 cycles = +++, 31-40 cycles = ++, > 41 cycles = +

Listed in this table are all strains tested by quantitative PCR for type-specific BoNT. All serotype primer and probe sets were tested against all strains indicated. Standards indicate the plasmid standards used to determine the quantity of BoNT DNA in each sample. Strains tested on the ABI 7700 machine (ABI) included purified DNA from bacterial cultures while samples tested by the Roche Light Cycler (LC) were from crude toxin supernatants.

With the same DNA preparations described in the previous section from healthy infant stool spiked with *C. botulinum *DNA, we were able to detect type-specific BoNT DNA reliably within all samples spiked with BoNT DNA at the equivalent of 10,000 genomic copies. The stool sample from the confirmed case of infant botulism yielded a positive result with 1650 BoNT/A specific gene copies detected in 5 μL of DNA extracted from the stool sample (Table 7). This confirms the result that had been obtained in the mouse protection bioassay that had been performed for clinical diagnosis.

**Table 7:** BoNT DNA detection in spiked healthy infant stool and botulism clinical samples

Spiked healthy infant stool	BoNT A	+ 5525
	BoNT B	+ 7179
	BoNT C	+ 234
	BoNT D	+ 187
	BoNT E	+ 4043
	BoNT F	+ 604
	BoNT G	+ 219
	None	-
		
Stool sample from clinical infant botulism case	BoNT A	+ 1650
	BoNT B	-
	BoNT C	-
	BoNT D	-
	BoNT E	-
	BoNT F	-
	BoNT G	-

DNA extracted samples were tested by real time quantitative PCR (qPCR) for detection and copy number of each BoNT serotype. Shown are results from approximately 10^4 ^genomic copies of DNA into each spiked sample prior to DNA extraction. (+) indicates a positive result with BoNT DNA copy number indicated in brackets. (-) indicates no amplification.

Listed in this table are the three conditions we tested for serotype-specific BoNT DNA from spiked healthy infant stool and a clinical sample of a confirmed case of infant botulism. For healthy infant stool, shown are results from samples spiked with BoNT DNA with 10^4 ^genomic equivalents. The clinical sample was run without dilution. (+) indicates a positive result and the copy number calculated from standard curves specific to each serotype is indicated in brackets. (-) indicates no amplification.

## Discussion

The spectre of bioterrorist use of botulinum toxin presents a new and real danger to public health [4,41], and in such an event a sensitive, specific and rapid diagnostic assay to detect the presence of the bacterium and/or its toxin will be needed. In addition, the possibility of botulinum toxin contamination of manufactured food requires constant monitoring. Indeed, over 90 different food products have been recalled in 2007 due to botulinum toxin contamination http://www.cdc.gov/botulism/botulism.htm. The current gold-standard assay, the mouse protection bioassay, is impractical in situations needing high-throughput analysis of multiple samples possibly at multiple geographical locations. In 2003 the National Institute of Allergy and Infectious Disease (NIAID) issued recommendations for new assays needed to detect botulism (NIAID Expert Panel on Botulism Diagnostics, Bethesda Maryland, May 2003). These recommendations stated that any new assay should be "universal", should be able to detect variants of all toxin types, should be type-specific to determine proper antitoxin treatment, and should be sensitive and quantitative to determine risk assessment.

Various methods that have been reported to address these requirements include immunological assays such as ELISA, ECL western blotting and Immuno-PCR, enzymatic assays such as EndoPEP assays and molecular techniques such as PCR [42-47]. The assays developed thus far offer a more rapid means of diagnosing botulism, but each also has limitations in such areas as sample throughput, cost, inability to distinguish toxin types, ease of use and false negative results [18,48].

PCR is a valuable methodology because it is sensitive, specific, cost-effective, portable, automatable, and high-throughput. However, PCR methods have certain limitations, such as the inability to distinguish between biologically active toxin genes and silent toxin genes in the bacterium [18]. While this is an important limitation as it is the protein toxin rather than the DNA encoding it that poses the threat, this is a rare occurrence since complete loss of toxicity in *C. botulinum *strains is usually accompanied by loss of phage or plasmids that contain toxin complex genes (personal observations of the co-authors) [49-51]. However, the consistent presence of *C. botulinum *DNA in even highly purified toxin preparations can serve as a surrogate marker and indicate the presence of toxin when *C. botulinum *contamination is suspected (T. Smith, unpublished data). Several different PCR methods have been reported, ranging from conventional electrophoresis-based PCR, including multiplex PCR, to real-time PCR and probe hybridization [20,23,27,28,38,48,52,53]. Each PCR-based method is reportedly faster and cheaper than the standard mouse protection bioassay [23]. However, most PCR assays detect a narrow range of toxin types, notably A, B, E and/or F, and do not consider the known genetic variation (subtypes) within each particular toxin type [32,33,54,55]. Botulinum neurotoxins, and their genes, exhibit an extreme amount of variability. Currently, there have been over 26 toxin subtypes identified. These toxin subtypes vary by ~1-32% at the amino acid level and their genes vary by approximately the same percentage at the nucleotide level. Despite this variability, we have been able to successfully detect and correctly identify the serotypes from samples representing 22 of these toxin subtypes.

In our study, we also use PCR technology to detect BoNT DNA in samples attempting to match the mouse protection bioassay in sensitivity and specificity. Our results show that we do surpass the sensitivity and specificity of the mouse protection bioassay in purified DNA when parallel samples of known toxicity and/or BoNT serotype are tested.

We detect BoNT DNA in samples reliably down to ten genomic copies in all strains of each subtype tested. In addition, our assay identified both toxins associated with our bivalent strains, while initial testing using the mouse bioassay only identified the predominant toxin in each case. The PCR assay also differentiated mosaic C/D and D/C strains from parental C and D strains; other methodologies are unable to differentiate these subtypes. With respect to the lower sensitivity of BoNT E detection, the data suggest that the initial genomic load of BoNT E DNA was lower than that of other subtypes. Based on the sensitivity of the assay presented here, BoNT E DNA of the same initial genomic load as the other subtypes tested will exhibit the same sensitivity surpassing the mouse protection bioassay.

Based on previous work to detect the presence of microbial 16S ribosomal DNA in human plasma samples during human immunodeficiency virus (HIV) infection to determine microbial translocation, we were able to determine the presence of bacterial DNA in human plasma using similar extraction and quantitative PCR techniques as described here [56]. Clearly, when dealing with clinical samples such as stool in which PCR inhibitors may present a challenge in detection of the BoNT DNA genes, there was a decrease in the detection limit of spiked healthy infant stool sample. However, in testing a confirmed infant botulism case in which the DNA tested was obtained from stool, we were readily able to determine the presence of the NTNH gene as well as its type and concentration.

## Conclusions

The two-step PCR assay described here fulfils the criteria recommended by the NIAID expert panel [57]. The first step, universal PCR detects the NTNH toxin complex gene that is conserved in all *C. botulinum *strains. The NTNH gene can be used as a high-throughput screening tool to determine those samples or individuals contaminated or infected with *C. botulinum *regardless of the type. The second step qPCR is used to determine the specific toxin type present and to estimate the extent of contamination by determining the gene load in each sample. A measure of the BoNT gene load may be helpful to the food industry to detect the presence and extent of contamination. Although the BoNT gene load may not predict the severity of illness, a fast, sensitive, and specific toxin detection assay will enable prompt administration of appropriate antitoxin therapy and assessment of the public health risk from suspect foods. With this assay's high-throughput capability and its portability, any laboratory may use it to assess quickly the possible presence of BoNTs either in food processing samples or in suspected cases of botulism. Thus, this assay provides rapid and specific detection of BoNT and toxin complex genes and would enable the targeting of appropriate therapeutic agents (eg: BabyBig^® ^or equine antitoxin) to infected individuals in a timely manner.

## Methods

### Bacterial strains and DNA purification

All strains tested within this report are listed in Table 8. DNA used in each PCR test was extracted from bacterial cultures as previously described [32]. Briefly, TPGY broth (Difco, Becton Dickinson and Co., Franklin Lakes, NJ) was inoculated with isolated *C. botulinum *bacterial colonies from each type and incubated anaerobically for 48 hours at 35°C for Group I strains and Group II strains were grown at 30 C°C followed by low speed centrifugation harvesting. The pellets were resuspended in TE and quickly frozen in a dry ice/ethanol bath at -70°C for three successive cycles followed by melting at 65°C. Sodium dodecylsulfate (SDS) and Proteinase K (10 mg/ml) were added, mixed, and incubated at 42°C for 1 hour. After incubation, 5 M NaCl solution and 10% (w/v) CTAB (cetyl trimethyl ammonium bromide) solution were added, mixed thoroughly and incubated at 65°C for 10 minutes. Following this incubation, three organic extractions of the mixture were performed using phenol/chloroform/isoamyl alcohol. DNA concentration was measured by spectrophotometry and diluted to a concentration of 25 μg/mL.

**Table 8:** Bacterial strains tested in PCR

	serotype	toxin type produced	strain
C. botulinum	A	A1	Hall
C. botulinum	A	A1	CDC 1757 (infant)
C. botulinum	A	A1	CDC 1744 (infant)
C. botulinum	A	A2	Kyoto-F (infant)
C. botulinum	Ab	A2b	CDC 1436 (infant)
C. botulinum	A	A3	Loch Maree
C. botulinum	B	B1	Okra
C. botulinum	B	B1	CDC 1656 (infant)
C. botulinum	B	B1	CDC 1758 (infant)
C. botulinum	B	B2	213B
C. botulinum	B	B2	CDC 1828 (infant)
C. botulinum	B	B3	CDC 795
C. botulinum	B	B4 (npB)	Eklund 17B
C. botulinum	Ba	Ba4	CDC 657 (infant)
C. botulinum	Bf	Bf	An436 (infant)
C. botulinum	C	C	Stockholm
C. botulinum	C	C/D	6813
C. botulinum	D	D	ATCC 11873
C. botulinum	D	D	1873
C. botulinum	D	D/C	VPI 5995
C. botulinum	E	E1	Beluga
C. botulinum	E	E2	CDC 5247
C. botulinum	E	E2	CDC 5906
C. botulinum	E	E3	Alaska E43
C. butyricum	E	E4	BL5262 (infant)
C. botulinum	F	F1 (prot)	Langeland
C. botulinum	F	F2 (np)	Eklund 202F
C. baratii	F	F3	Orange
C. botulinum	G	G	1354
C. absonum			ATCC 27555
C. baratii			ATCC 27638
C. bifermentans			ATCC 638
C. haemolyticum			ATCC 9650
C. hastiforme			ATCC 25772
C. histolyticum		histolyticum α, β	ATCC 19401
C. novyi			ATCC 17861
C. novyi			ATCC 19402
C. novyi	A	novyi α, γ, ε	ATCC 19402
C. novyi	B	novyi α, β	ATCC 2706
C. perfringens	A	perfringens α	ATCC 3624
C. perfringens	A	perfringens α	ATCC 12915
C. perfringens	A	perfringens α	ATCC 12917
C. perfringens	A	perfringens α	ATCC 12918
C. perfringens	A	perfringens α	ATCC 12919
C. perfringens	A	perfringens α	ATCC 13124
C. perfringens	B	perfringens α, β, ε	ATCC 3626
C. perfringens	D	perfringens α, ε	ATCC 3629
C. perfringens	D	perfringens α, ε	ATCC 3630
C. perfringens	D	perfringens α, ε	ATCC 3631
C. perfringens	D	perfringens α, ε	ATCC 12920
C. perfringens	E	perfringens α, τ	ATCC 27324
C. ramosum			ATCC 25582
C. septicum		septicum α	ATCC 12464
C. sordelli			ATCC 9714
C. sporogenes			ATCC 19404
C. sporogenes			ATCC3854
C. subterminale			ATCC 25774
C. tertium			ATCC 14573
C. tetani		tetanus	ATCC 10799
C. tetani		tetanus	ATCC19406

*The C. botulinum *and BoNT E-producing *C. butyricum *strains are from the USAMRIID

*C. botulinum *culture collection, which forms part of the Unified Culture Collection.

The other Clostridium species were obtained from ATCC.

**All clostridial species tested in these studies are listed with strain identifications. Where applicable toxin serotype and/or toxin types are shown**.

### Crude Toxin Supernatant Preparation

Isolated colonies from an egg yolk or blood agar plate that had been incubated for 48 hours in a gas pack jar were inoculated in ten mL of TPGY broth, (5% Trypticase, 0.5% Bacto Peptone, 2% Yeast extract, 0.4% glucose and 0.2% Cystene). The TPGY broth was then incubated for 5 days at 35°C for proteolytic cultures and 30°C for non-proteolytic cultures in a gas pack jar. Samples were then centrifuged at 4000 rpm for 15 minutes and supernatant was filtered through a 0.22 μm membrane filter. Aliquots were made and stored at -70°C until needed. Sample sterility was tested on blood agar plates that were incubated for 48 hrs then checked for growth.

### DNA extraction from spiked food, healthy infant stool, crude toxin samples and infant botulism clinical sample

Canned vegetables and meat from a local market and stool from a healthy infant were separated into aliquots of 200 mg amounts of material. Each solid aliquot was homogenized using a mortar and pestle into a paste. 100 μL of purified DNA from specific *C. botulinum *strains was added to the food or stool paste at dilutions ranging from 10^5 ^to 10 genomic copies. DNA from each sample was then extracted using Qiagen's QiAMP DNA stool mini kit (Qiagen, Valencia CA) using manufacturer's recommendations with one modification. Each sample was bound to the column provided in the kit and washed twice before proceeding to further steps to ensure elimination of any protein debris that may interfere with subsequent PCR analysis. For crude toxin supernatants, DNA was extracted from 200 μL of crude supernatant using the QiAmp DNA stool mini kit as described above. For spiked food, healthy infant stool samples and crude supernatants, extracted DNA was eluted in 50 μL of elution buffer and immediately tested for presence of either NTNH or type-specific BoNT. NTNH assays were done on DNA extracted from crude culture supernatants, as outlined above. The BoNT serotype-specific assays were done on crude culture supernatants with no further extraction or processing. Infant stool DNA from a clinical sample was extracted with the MagNA Pure compact instrument and Nucleic Acid Isolation Kit I (Roche Applied Science) according to the manufacturer instructions.

#### Universal PCR primer designed for NTNH

Sequences used to design a set of universal PCR primers were obtained from Genbank. All sequences were aligned with MegAlign (DNASTAR, Lasergene, Inc.). Both Primer Express (Applied Biosystems, Foster City, CA) and Primer3 (http://frodo.wi.mit.edu/primer3/), were used to design a pair of degenerate primers that included base differences to detect all known NTNH gene variants. Primer sequences are designated in Figure 1.

#### Universal PCR for detection of NTNH of all *C. botulinum *types

Purified DNA from *C. botulinum*, *E. coli *bacterial DNA (pUC19 plasmid DNA) or crude lysate from human leukocytes were used in the universal PCR. PCR conditions were as follows: 95°C for 5 minutes, then 35 cycles of 95°C for 15 seconds and 57°C for 1 minute. PCR reaction mixture contained PCR Buffer, 3.5 uM MgCl_2_, 200 nM dNTP, 1 uM forward or reverse primer, 0.25 U Taq Polymerase (Invitrogen Corp, Carlsbad, CA). 5 μL of DNA (0.25 ng/uL) was used in each 25 μL PCR reaction. PCR products were run on a 2.5% agarose gel to separate the product from any non-specific amplification and visualized for 101 bp bands by UV illumination.

#### Toxin type-specific qPCR primer and probe design

Neurotoxin gene sequences, obtained both from Genbank and from sequences provided by Biosciences Division, Los Alamos National Laboratories, were aligned and degenerate primer/probe sets were designed using software packages as above for each toxin type. Each degenerate primer/probe set include all known base differences within each toxin type.

#### Generation of qPCR standards for each *C. botulinum *toxin type-specific assay

Seven samples of purified *C. botulinum *DNA, one for each toxin type, were used in the generation of plasmid DNA standards for qPCR. Briefly, primers designed specifically for each toxin type were used to amplify a region of the toxin gene containing the degenerate primer/probe set target sequences. The PCR conditions were as follows: 95°C for 5 minutes, then 35 cycles of 95°C for 15 seconds and 60°C for 1 minute. PCR reaction mixture contained PCR Buffer, 3.5 uM MgCl_2_, 200 nM dNTPs, 500 nM forward or reverse primer, 0.25 U Taq Polymerase (Invitrogen). 5 μL of DNA (0.25 ng/uL) was used in each 25 μL PCR reaction. PCR products were visualized by UV on a 1.5% agarose gel. Corresponding specific products were gel purified and ligated into pGEM T-easy vector (Promega Corp., Madison, WI). Ligations were transformed into DH5α *E.coli *bacteria using α-complementation to determine positive colonies. Positive colonies were grown in overnight cultures, plasmid DNA was purified and sequenced for determination of correct subtype insert sequence. DNA was quantified by UV spectrophotometry and serial dilutions of plasmid standard were made from 10^6 ^to 10^2 ^target molecules/5 μL.

#### qPCR for BoNT Type-Specific Detection

The qPCR assay consisted of seven separate reactions, each specific for one of the seven neurotoxin gene types. For absolute quantification, template standards for each of the neurotoxin gene types were run alongside the DNA samples for each of the seven qPCRs. qPCR conditions were as follows: 95°C for 5 minutes, then 45 cycles of 95°C for 15 seconds and 60°C for 1 minute. PCR reaction mixture contained PCR Buffer, 3.5 uM MgCl_2_, 200 nM dNTPs, 500 nM forward or reverse primer, 200 nM Fam/BHQ1-labeled probe, 3 nM BD636 reference dye, 0.25 U Taq Polymerase (Invitrogen Corp, Carlsbad, CA). 5 μL of purified DNA or plasmid standard was used in each 25 μL PCR reaction. Based on cycle of threshold (Ct) values with known copy numbers of plasmid in each reaction, a standard curve is generated that will be used to calculate the values of unknown samples.

## Authors' contributions

BH designed all primers and probes and optimized and performed PCRs based on purified DNA or spiked food samples as well as clinical samples. JS performed all PCR assays on crude toxin preparations. TS provided DNA and crude toxin preparations for PCR testing. DD and SA conceived the study and guided its design. All authors contributed to interpretation of data and preparation of this manuscript. All authors have read and approve of this final manuscript.
